# Ex-vivo validation of nine algorithms for quantifying infarcts with late gadolinium enhancement cardiovascular magnetic resonance

**DOI:** 10.1016/j.jocmr.2025.101915

**Published:** 2025-05-29

**Authors:** Sascha Kopic, Einar Heiberg, Henrik Engblom, Marcus Carlsson, David Nordlund, Robert Jablonowski, Mikael Kanski, Christos Xanthis, Sebastian Bidhult, Anthony H. Aletras, Håkan Arheden

**Affiliations:** a Lund University, Department of Clinical Sciences Lund, Clinical Physiology, Skane University Hospital, Lund, Sweden; b Lund University, Wallenberg Center for Molecular Medicine, Lund, Sweden; c School of Medicine, Aristotle University of Thessaloniki, Laboratory of Computing, Medical Informatics and Biomedical – Imaging Technologies, Thessaloniki, Greece

**Keywords:** Late gadolinium enhancement, Infarct size, Quantification, Accuracy, Precision

## Abstract

**Background:**

In cardiovascular magnetic resonance, late gadolinium enhancement (LGE) is the standard method to visualize myocardial infarction (MI). Many algorithms quantifying infarct size in LGE images exist. However, only few algorithms have been validated, i.e., benchmarked against an ex-vivo measurement. Furthermore, the reported algorithm performance varies considerably between studies.

**Objectives:**

The aim of this study was to compare the performance of all infarct measurement algorithms against an ex-vivo measurement and to promote a discourse regarding advantages and disadvantages of individual measurement methods.

**Methods:**

MI was induced in 22 pigs. In-vivo LGE imaging was conducted on d0, d3 or d7 post-MI. For ex-vivo validation infarct was measured using high-resolution T1-weighted images. In-vivo infarct size was measured using the full-width at half-maximum (FWHM), *n*-SD from remote (2,3,5, and 6 SD), feature analysis and combined thresholding (FACT), expectation maximization-weighted A priori information (EWA), Heiberg-08 and Otsu algorithms and manual delineation. No manual adjustments were made to algorithm delineations.

**Results:**

Clear differences in variance and bias were observed between algorithm-based methods, and no method performed optimally in this heterogeneous dataset where the best had a bias of −0.48 ± 3.1, −0.3 ± 4.4%, 2.3 ± 4.2% left ventricle for EWA, FWHM, and FACT, respectively. Manual delineation by experienced observers performed well with a bias of 1.9 ± 5.4%.

**Conclusion:**

EWA, Heiberg-08, FWHM, and FACT all perform on par with manual delineation, however, Heiberg-08, and FWHM are not suitable for phase sensitive inversion recovery images. The technique used to measure infarct size should be disclosed in clinical trials and in original research. Caution should be applied when comparing datasets employing different infarct quantification methods. Manual infarct delineation by experienced readers remains a reliable technique to measure infarct size.

## Graphical abstract

**Figure:**
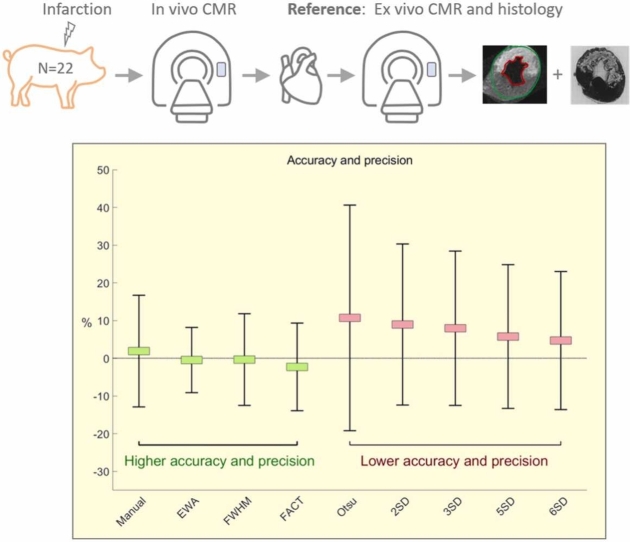
Central illustration: Out of nine algorithms for infarct quantification on late gadolinium enhancement three had accuracy and precision on par with manual delineation when evaluated against high resolution T1w ex-vivo images. Estimated bias is indicated with a box and the precision is indicated by with bars and show the limits of agreement. The methods that performed acceptably were EWA (Expectation maximization Weighted A priori information), FWHM (Full Width Half Maximum), and FACT (Feature Analysis and Combined Thresholding).

## 1. Introduction

Cardiovascular magnetic resonance (CMR) has become an increasingly important method for the characterization of myocardial infarction (MI). The reference standard for infarct size measurement with CMR is late gadolinium enhancement (LGE) imaging [1]. LGE sequences visualize T1-weighted image contrast by leveraging the volume distribution of the extracellular contrast agent between areas with high extracellular volume fractions, such as necrotic or scarred myocardium, and viable myocardium [2]. The ability to accurately quantify MI has led to a rise of the utilization of CMR in clinical routine, but also in clinical trials [3], [4].

While protocols to capture optimal LGE-based contrast have been standardized, manual measurement of the infarcted area relies on subjective parameters, such as the reader’s experience level. This poses a challenge for interpretation of data in multi-center trials or even between readers within the same institution.

To address the potential for subjective bias, several groups have proposed algorithms that assign pixels in LGE-based images to either infarcted or non-infarcted. The algorithms operate either in a fully automated or semi-automated fashion, requiring some degree of user input. Automated or semi-automated algorithms have the potential of limiting subjective bias, but also to reduce the time needed for infarct size analysis. However, only a subset of the algorithms is experimentally validated, i.e. tested against an ex-vivo measurement standard (for an overview of common algorithms see Table 1).

**Table 1:** Comparison of infarct algorithms.

Algorithm name	Brief description	Experimental validation	Imaging time-point post-MI	Ex-vivo validation technique	Necessity for manual interaction*	LGE sequence	MVO detection
Full-width at half-maximum (FWHM) [8]	1. User provides seed point in suspected infarct area on each slice 2. Multi-pass region growing algorithm applied based on FWHM criteria 3. Note: FW defined as: 0(min) – Max PI(max)	Yes 13 mongrel dogs	d0	TTC	Yes User provides seed point in suspected infarcted area	IR	No
Feature analysis and combined thresholding (FACT)[18]	1. Initial thresholding 2 SD above mean of remote to identify potential infarct areas 2. Feature analysis: Removal of false positive areas based on volume (min volume 0,1 g), distance to endocardium (max. 2 mm from endocardium) and mean intensity (intensity above 50% of average of all pot. infarct areas) 3. FWHM threshold applied within pot. infarct areas 4. Note: contrarily to (11) FW defined as: Average of remote PI(min) – Max PI(max), not 0(min) – Max PI(max) 5. Repeat step 2 6. Inclusion of MVO (defined as dark areas surrounded either by infarct or endocardium)	Yes 11 mongrel dogs	d2 (n=5), m2 (n=6)	TTC	No	PSIR	Yes
*n*-SD from remote [10]	1. User defines remote myocardium (with setting of ROI) 2. Determination of infarcted pixels as mean PI_remote_ + x SD	Yes (3 SD from remote) 28 mongrel dogs	d1, d3, d10, w4, w8	High-res T1 weighted; TTC	Yes Remote and potentially scar ROIs have to be defined	IR; works with PSIR	No
Heiberg et al. 2008 [16]	1. Segmentation of ventricular short-axis slice into 5 sectors 2. Midmural half of sector with lowest mean SI defined as remote myocardium 3. 1.8-SD from remote thresholding 4. Exclusion of dis-contiguous artifacts by means of a level set method (29) 5. Removal of isolated volumes < 1.5 cm^3^ 6. Linear weighting depending on pixel intensity	Yes 8 pigs	d0	High-res T1 weighted	No	IR	Yes
Expectation maximization, Weighted intensity, A priori information (EWA) [17]	1. Surface coil intensity correction 2. Classification of myocardial intensities by means of a constrained EM-algorithm (30): iterative refinement to find the maximum likelihood estimate of the mean and SD for the Gaussian PI distributions of normal myocardium and myocardial infarction 3. Exclusion of dis-contiguous artifacts by means of a level set method (29) 4. Inclusion of MVO 5. Post processing to exclude artifacts 6. Calculation of the infarct size by weighting the pixels based on their intensity	Yes 30 pigs	d0, d7	High-res T1 weighted; TTC	No	IR and PSIR	Yes
Otsu auto threshold (OAT) [20]	Algorithm assumes existence of two classes of pixels following bimodal distribution (infarct and remote) and calculates threshold based on minimized intra class variance and maximized inter class variance	No	N/A	N/A	No	IR; works with PSIR	No

*MI* myocardial infarction, *LGE* late gadolinium enhancement, *MVO* microvascular occlusion, *FWHM* full-width at half-maximum, *PI* pulsatility index, *TTC* triphenyltetrazolium chloride, *IR* inversion recovery, *PSIR* phase sensitive inversion recovery, *FACT* feature analysis and combined thresholding, *ROI* region of interest, *EWA* Expectation maximization Weighted A priori information, *OAT* Otsu auto threshold, *LV* left ventricle

* except delineation of LV contours

The reported performance of the algorithms varies considerably between studies. Rather than leveraging an ex-vivo measurement standard, most comparative reports employ manual infarct delineation as a benchmark [12], [13], [14]. In a meta study on prognostic value of LGE in dilated cardiomyopathy two thirds of the studies (21/34) used visual analysis where the remainder used different n-SD [15]. The abundance of available algorithms and the sometimes conflicting data regarding their performance is acknowledged by the Society for Cardiovascular Magnetic Resonance, which states in its consensus that as “evidence is being accumulated, the Task Force chooses to refrain from making a dedicated statement at this time regarding the optimal method for quantitative assessment” [9]. Somewhat disturbingly, it has been reported that in 27% of clinical trials utilizing CMR, the measurement method of infarct size is not specified at all [3].

In the fragmented landscape of infarct quantification and scarcity of ex-vivo validation, this study aims to compare the performance of commonly utilized infarct measurement algorithms against an ex-vivo reference standard in a porcine model of MI and to provide insights into advantages and disadvantages of each algorithm. The dataset used in this study intentionally represents a heterogeneous population including images acquired at various time points post MI and on 1.5T scanners from two different vendors, but also infarcts of various morphologies.

The aim of this study is to investigate performance of all different approaches for ischemic infarct quantification, and importantly also to promote a critical discourse by highlighting the challenges of each individual approach that need to be collectively overcome on the path toward the optimal algorithm.

## 2. Materials and methods

### 2.1. Experimental protocol

Pigs (n = 22; weight: 40–50 kg; gender: mixed) were anesthetized and subjected to occlusion of the left anterior descending (LAD) artery for 35–40 min, using a balloon catheter. The balloon occlusion was placed either distally of the first or second diagonal artery. Following reperfusion, animals were either imaged on the same day (approximately 6 h post reperfusion; n = 10), on day 3 (n = 6) or on day 7 (n = 6). The study was approved by the regional ethics committee for animal experiments.

### 2.2. CMR imaging in vivo

CMR imaging was performed on a 1.5T scanner (Philips Achieva, Best, Netherlands with a 32-channel cardiac coil or Siemens Magnetom Aera, Erlangen, Germany with a 18-channel receiver coil optimized for CMR applications [body array and spine array]).

Approximately 15 min prior to LGE imaging 0.2 mmol/kg gadolinium-tetraazacyclododecane-tetraacetic-acid (Gd-DOTA, Dotarem, Guerbet, Roissy, France) was administrated intravenously. Pulse sequences were optimized for the respective scanner vendor hardware. Two types of LGE images were acquired for all animals: inversion recovery (IR) and phase sensitive inversion recovery (PSIR) images. The PSIR images were reconstructed so that the sign of the magnetization was preserved. The slice was 8 mm with no slice gap for all images. Manual inversion time adjustment was performed to null the signal of viable myocardium.

On Philips (n = 16). The IR images were acquired using a 3D-IR sequence where the echo time was 1.3 ms, flip angle 15 degrees, slice thickness 8 mm and typical in plane resolution of 1.5 × 1.5 mm. The PSIR images were acquired using a 2D PSIR sequence with a flip angle of 25°, and typical resolution of 1.6 × 1.6 mm. This data were included from a previously published study [7].

On Siemens (n = 6), the IR images were acquired with echo time 1.21 ms and flip angle 45°and typical reconstructed image resolution 1.4 × 1.4 mm, PSIR images were acquired with echo time 1.18 ms, flip angle 50° and typical image resolution 1.4 × 1.4 mm. This followed the methodology in another previously published study [5].

### 2.3. CMR imaging ex-vivo

Following LGE imaging, another intravenous bolus of 0.2 mmol/kg Gd-DOTA was administered approximately 15 min before euthanasia. The heart was then immediately explanted and the ventricles were filled with balloons containing deuterated water. The filling was performed to avoid collapse of the ventricular walls. Thereafter, ex-vivo high-resolution T1-weighted (T1w) imaging was performed on the same scanner as the in-vivo imaging with a voxel size of 0.5 × 0.5 × 0.5 mm using a head coil. On Philips the sequence parameters were echo time 3.2 ms, repetition time 20 ms, flip angle, 70°; two number of signal averages. On Siemens the sequence parameters were repetition time 20 ms; echo time 3.6 ms; flip angle 70°. The T1w images were chosen as the reference standard as 1) it has significantly higher spatial resolution compared to the triphenyltetrazolium chloride (TTC) which has a slice resolution of at best 3 mm, and 2) TTC is challenging to measure volumetric information as the thin slices are deformed during the staining process.

### 2.4. TTC staining and image acquisition

For a subset of the animals, histopathological assessment of infarct size was performed. The hearts were cast in a solution of 2% agarose (SigmaAldrich, Stockholm, Sweden) and sliced into 5 mm thick slices that were stained with 5–6 g TTC (Sigma Aldrich, Stockholm, Sweden) dissolved in 0.5 L buffer (pH = 8.5) for 5 min at 35–37 °C. Finally, slices were photographed on both apical and basal sides for infarct analysis. For each heart, 8–10 slices were positive for infarction.

Image analysis was conducted using the software Segment (Medviso, Lund, Sweden). The software is freely available for research use and CE-marked and FDA 510(k) cleared for clinical usage [16]. The endocardium and epicardium were manually delineated on T1w images. A manual threshold was applied inside the myocardium corresponding to the observer’s visual impression of the infarct extent. A corresponding process was performed for the TTC images. Manual corrections were added if needed. For the LGE images, LV contours (endo- and epicardium) including papillary muscles were delineated by two expert readers with 26 and 20 years of experience. The first expert delineated the Philips images, and the second expert delineated the Siemens images.

The following algorithms and methods were used to quantify in-vivo infarct size: full-width at half-maximum (FWHM) [8], *n*-standard deviations (SD) from remote myocardium (2,3,5 and 6 SD) [10], Expectation maximization-weighted intensity-A priori information (EWA) [17], feature analysis and combined thresholding (FACT) [18], Heiberg-08 [19], Otsu method [20] and manual delineation. For an overview of general principles and pre-requisites of the algorithms, and how they have been validated, please see Table 1. The automated algorithms use different amounts of manual input. The following manual steps was performed:

• The LV contours were manually outlined.
• An *infarct region of interest (ROI)* was defined on each short axis image where infarct was visually identified or suspected. The infarct ROI was automatically cropped to not go outside the manually delineated LV contour.
• A *remote ROI* was drawn (typically in a semi-lunar shape) with the aim of maximizing the included area of viable myocardium.

The ROIs were drawn by a single observer and care was taken to avoid artifact regions to minimize potential bias.

The manual information was used by the different algorithms as follows:

• For n-SD algorithms, the remote ROI was used to compute a threshold that subsequently was applied inside the infarct ROI. Note that using this method the user could avoid artifact regions outside the infarct region.
• FACT algorithm only used the LV contours.
• For FWHM the LV contour and the *infarct ROI* was used, and the algorithm identified the maximum signal intensity within the infarct region. This represents a variation of the originally published algorithm, in which a single seed point is manually provided by the user in the suspected infarcted area and the algorithm subsequently autonomously grows an *infarct ROI* within which the highest signal intensity is identified (Table 1).
• Both EWA and Heiberg-08 only used the LV-contours.
• Otsu only used the LV contours.

**Fig. 1:**
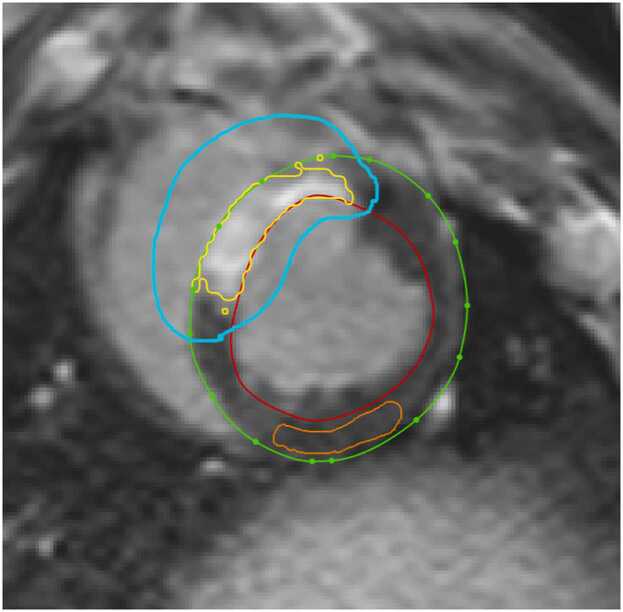
Example on ROIs used. The cyan color ROI defines the myocardium of interest in which the scar region (yellow) is delineated using the FWHM method. The n-SD method uses both the cyan ROI and the remote region ROI (orange) for infarct delineation. *ROI* region of interest, *FWHM* full-width at half-maximum

An example on how the ROIs for FWHM and n-SD from remote looks like are illustrated in Fig. 1. Note that all algorithms implicitly used the LV contours as the infarct was expressed as percentage of LV mass.

Manual infarct delineation was conducted by the same readers who delineated left ventricular contours. Readers were blinded to all information pertaining to the particular dataset. Two experienced observers (HA) and (HE) performed inter-observer analysis on 50% of the animals (N=12).

The reference infarct size was measured on ex-vivo high-resolution T1w images using a manual set threshold to delineate the infarcted area. The threshold was set individually for each animal by consensus between five expert readers with the aim of optimally reflecting the infarcted area. For each animal, the experts determined if the infarct was patchy or not in consensus (Fig. 7). TTC images were used as a visual comparator to inform optimal *n*-SD selection, particularly in cases of patchy infarction. For a subset of the animals, the ex-vivo T1w images were compared to manual planimetry of the TTC images. All these T1w images were acquired on a Philips scanner.

### 2.5. Data exclusion

Datasets with microvascular occlusion (MVO) were not included in this study. Two datasets were excluded from analysis for the n-SD from remote and FWHM algorithms, as no infarct ROI could be visually identified. In these cases, infarct size was set to zero, and these datapoints were still included in the statistical analysis. Consequently, both the n-SD from remote and FWHM algorithms were applied in 20 datasets.

### 2.6. Statistical methods

Infarct size was quantified for the whole heart and expressed as percentage of left ventricular mass. Bias and variability were analyzed using modified Bland–Altman analysis and plotted as difference in infarct size between methods against the high-resolution T1w- reference infarct size. In addition, concordance correlation coefficient or Lin coefficient was computed. T-test was used to check for statistically significant bias and compared to reference infarct size, and p-value less than 0.05 was considered to indicate significance. Performance of the automated algorithms was considered acceptable if the difference in infarct size compared to reference standard was on par with manual delineation. Given uncertainties in the data on par was defined as no more than 1% unit from manual delineation in both bias and variability. Statistics was computed with Matlab R2022a (Mathworks, Natick, Massachusetts).

## 3. Results

Table 1 gives an overview of all algorithms and their underlying assumptions and how they have previously been validated.

**Fig. 2:**
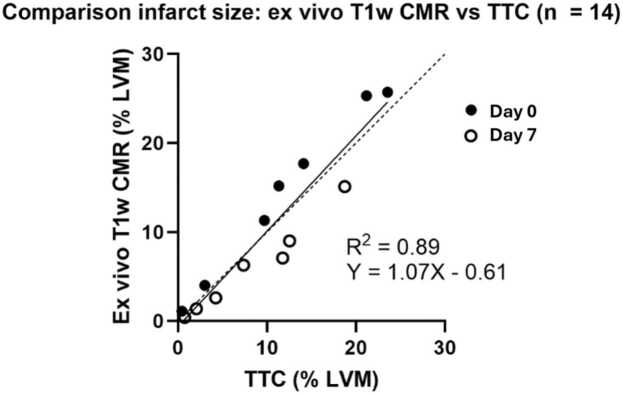
Comparison between high-resolution ex-vivo T1w CMR vs. TTC in a subset of the animals (n = 14) where TTC imaging was available. Filled circles indicate d0 and open circles indicate d7. *TTC* triphenyltetrazolium chloride, *CMR* cardiovascular magnetic resonance, *T1w* T1-weighted

**Fig. 3:**
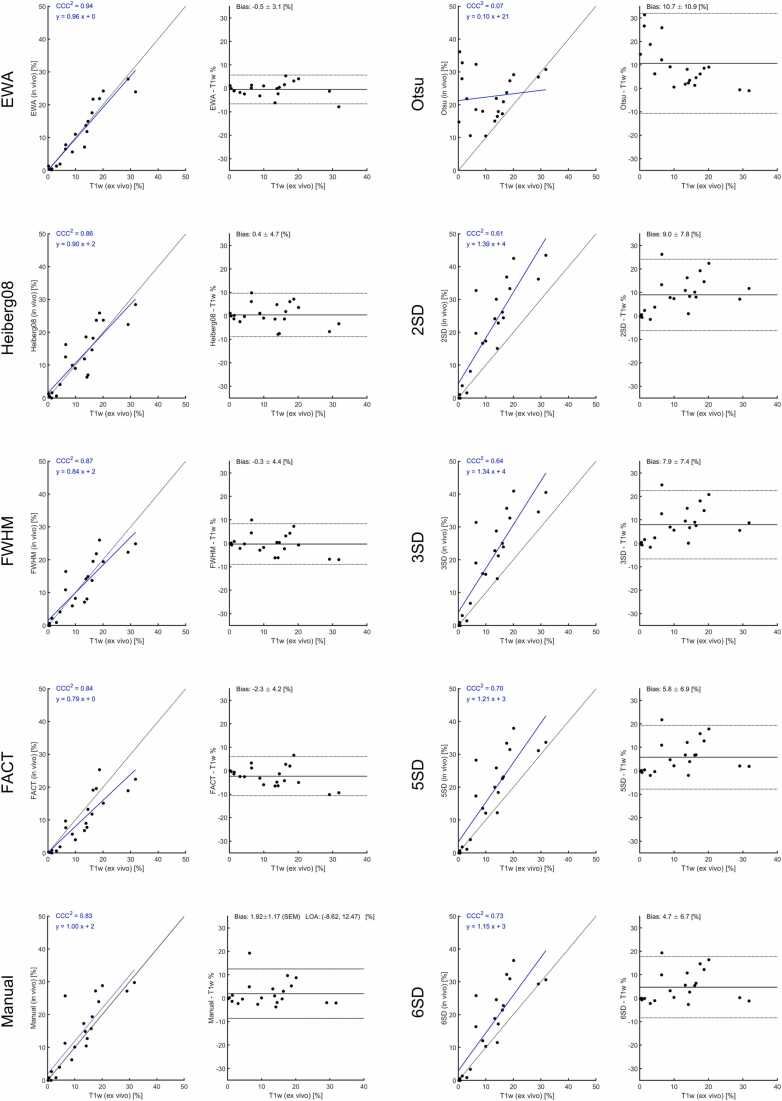
Agreement between IR in-vivo quantification methods and ex-vivo T1w high-resolution infarct measurements. Left panels show agreement between both measurements including identity line (dashed) and regression line in blue. Right panels show modified Bland–Altman blot plot including bias (solid line) and 95% limits of agreement in dashed lines. Bias in text gives bias ± SD. *IR* inversion recovery, *T1w* T1-weighted, *SD* standard devia

**Fig. 4:**
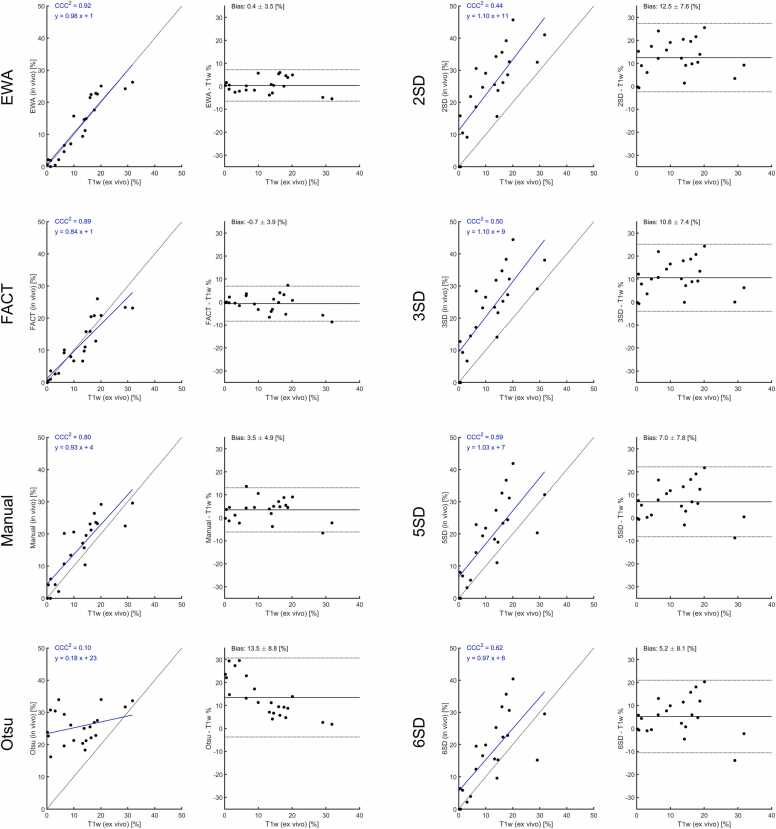
Agreement between PSIR in-vivo quantification methods and ex-vivo T1w high-resolution infarct measurement. Left panels show agreement between both measurements including identity line (dashed) and regression line in blue. Right panels show modified Bland–Altman blot plot including bias (solid line) and 95% limits of agreement in dashed lines. Bias in text gives bias ± SD. *PSIR* phase sensitive inversion recovery, *T1w* T1-weighted

Fig. 2 shows the comparison between ex-vivo high-resolution T1w CMR vs. TTC, revealing an excellent agreement between the two methods indicating that ex-vivo high-resolution T1w CMR can be used as reference standard in the current setting. The bias and variability (SD) for T1w CMR compared to TTC was 0.1 ± 2.8 (%-pts). The inter-observer variability of manual scar analysis on T1w images was 0.04 ± 2.3 (%-pts). The manual infarct size on T1w inversion recovery images was (mean ± SD) 14.2 ± 10.7%, 12,0 ± 8.8 mL. The range for the infarct size was 0–33%, and 0–27.3 mL. The mean transmurality for the animals with infarct was 62 ± 13%, range (38–80%), the maximum transmurality was 99 ± 4% (range 86–100%).Fig. 3 shows the agreement between in-vivo inversion recovery LGE infarct size, as measured by the different algorithms or manual delineation using the ex-vivo high-resolution T1w images as reference. Fig. 4 shows the data for PSIR images. The methods EWA, Heiberg-08, FWHM, and FACT all performed on par with manual delineation on IR images compared to T1w reference. The bias and variability (SD) of the methods were for IR images; −0.5 ± 3.1, 0.4 ± 4.7, −0.3 ± 4.4, −2.3 ± 4.2 (%-pts), for EWA, Heiberg-08, FWHM, and FACT, respectively. The bias and variability of manual delineation on IR images compared to T1w reference was 1.9 ± 5.4 (%-pts). EWA and FACT performed on par with manual delineation on PSIR images compared to T1w reference. The bias and variability (SD) of the methods were for IR images using EWA; 0.4 ± 3.5, and for FACT −0.7 ± 3.9 (%-pts). The bias and variability of manual delineation on PSIR images compared to T1w reference was 3.5 ± 4.9 (%-pts).

All n-SD from remote and Otsu overestimated the infarct size with high variability.

Bias, variability (SD), and 95% Limits of agreement as well as concordance correlation coefficient for all algorithms and manual delineation are summarized in Table 2.

**Table 2:** Data summary.

	CCC		Eq		Bias (mean ± SD) (%-pts) of LV mass		LoA (mean ± 1.96 SD) (%-pts) of LV mass			P-value	
*Method*	*IR*	*PSIR*	*IR*	*PSIR*	*IR*	*PSIR*	*IR*		*PSIR*	*IR*	*PSIR*
EWA	0.94	0.92	Y=0.96x +0	Y=0.98x +1	−0.5 ± 3.1	0.4 ± 3.5	[−6.6, 5.6]		[−6.5, 7.2]	0.51	0.64
Heiberg−08	0.86	-	Y=0.90x +2	-	0.4 ± 4.7	-	[−8.9, 9.6]		-	0.71	-
FWHM	0.87	-	Y=0.84x +2	-	−0.3 ± 4.4	-	[−9.0, 8.3]		-	0.74	-
2 SD from remote	0.61	0.44	Y=1.39x +4	Y=1.10x+11	9.0 ± 7.8	12.5 ± 7.6	[−6.3, 24.2]		[ −2.4, 27.4]	<0.01*	<0.01*
3 SD from remote	0.64	0.50	Y=1.34x +4	Y=1.10x+9	7.9 ± 7.4	10.6 ± 7.4	[−6.7, 22.5]		[−4.0, 25.2]	<0.01*	<0.01*
5 SD from remote	0.70	0.59	Y=1.21x +3	Y=1.03x +7	5.8 ± 6.9	7.0 ± 7.8	[−7.8, 19.3]		[−8.3, 22.2]	<0.01*	<0.01*
6 SD from remote	0.73	0.62	Y=1.15x +3	Y=0.97x +6	4.7 ± 6.7	5.2 ± 8.1	[−8.4, 17.8]		[−10.6, 21.0]	<0.01*	<0.01*
Otsu	0.07	0.10	Y=0.10x+11	Y=0.18x+23	10.7 ± 10.9	13.5 ± 8.8	[−10.7, 32.0]		[−3.7, 30.7]	<0.01*	<0.01*
FACT	0.84	0.89	Y=0.79x +0	Y=0.84x +1	−2.3 ± 4.2	−0.7 ± 3.9	[−10.6, 6.0]		[−8.3, 6.9]	0.02*	0.41
Manual	0.83	0.80	Y=1.00x +2	Y=0.93x +4	1.9 ± 5.4	3.5 ± 4.9	[−8.6, 12.5]		[−6.2, 13.1]	0.12	<0.01*

*LV* left ventricle, *EWA* expectation maximization Weighted A priori information, *FACT* feature analysis and combined thresholding, *IR* inversion recovery, *PSIR* phase sensitive inversion recovery, *CCC* concordance correlation coefficient or Lin coefficient. – Denotes that the method was not designed to work for PSIR images. LoA denotes limits of agreement computed as mean ± 1.96 SD. * Denotes that the bias is significantly different from zero compared to reference method

Numbers on the form xx +- yy represent mean +- SD, whereas numbers in brackets indicate LoA.

**Fig. 5:**
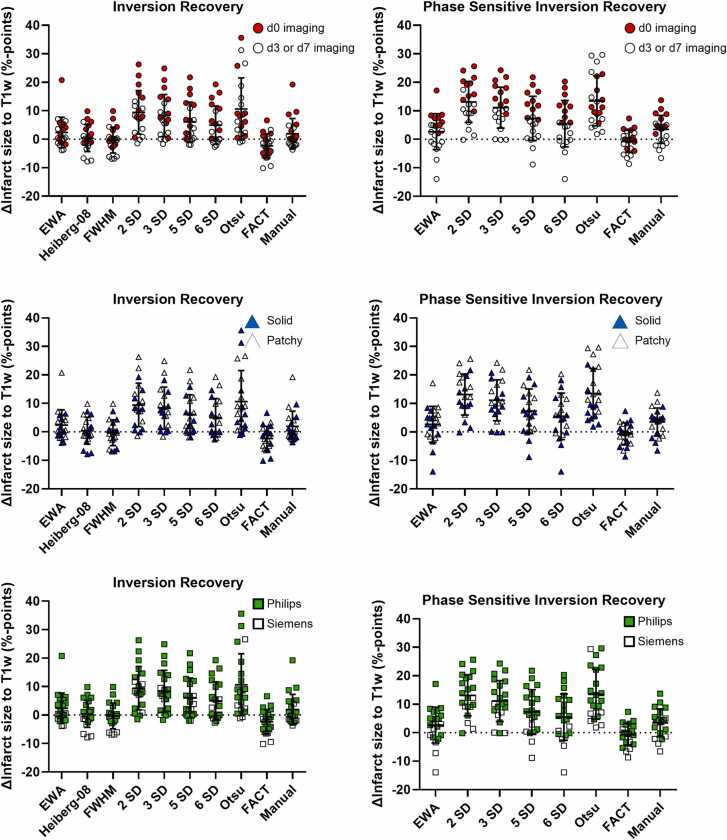
Comparison of bias and SD between infarct quantification methods. The left column shows data for inversion recovery and the right column shows data for phase sensitive inversion recovery. Top row highlights time point after infarction (d0 vs. d3/7). Middle row highlights infarct morphology (solid vs. patchy). The bottom row highlights scanner vendor. There is no clear trend that any method performs differently on different types of infarctions *EWA* expectation maximization Weighted A priori information, *FWHM* full width at half maximum, *SD* standard deviation, *FACT* feature analysis and combined thresholding

Fig. 5 provides more detailed information on the bias of each method including manual delineation. Imaging time point, type of infarction, and scanner type are indicated in the figure.

**Fig. 6:**
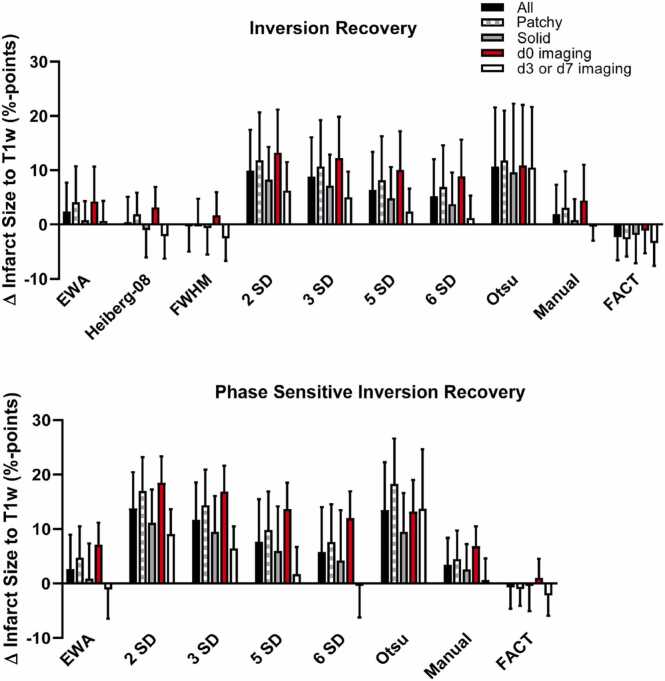
Comparison of bias and variability (SD) between infarct quantification methods. Data presented for different methods and infarct morphology (solid vs. patchy), and imaging time point (d0, vs. d3/7). *EWA* expectation maximization Weighted A priori information, *SD* standard deviation, *FACT* feature analysis and combined thresholding

Fig. 6 provides more detailed information on the bias and variability of each method including manual delineation. Imaging time point, type of infarction, and scanner type are indicated in the figure.

**Fig. 7:**
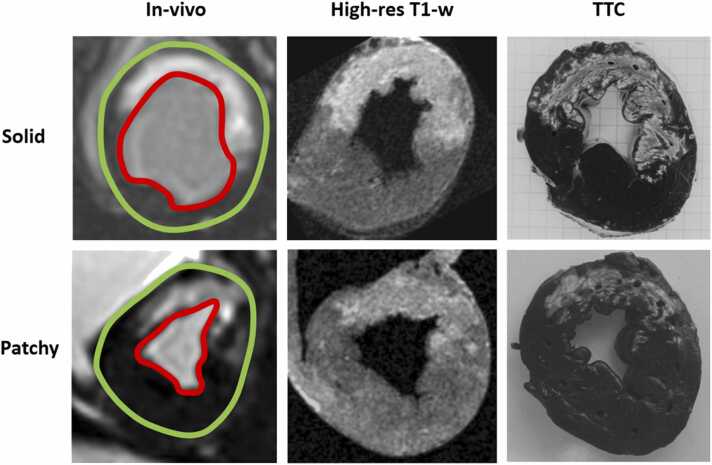
Comparison of solid and patchy infarctions. Top row shows a subject with solid infarction and the bottom row shows a patchy infarction. Left column shows in-vivo images where the endocardium is indicated in red and the epicardium in green. Middle column shows high-resolution T1w images used as reference standard and right column shows corresponding TTC images. Note that the patchy infarct shows a more insular infarction pattern, with areas of lower pixel intensity in both T1w and in-vivo LGE images. *TTC* triphenyltetrazolium chloride, *T1w* T1-weighted, *LGE* late gadolinium enhancement

Overall, the EWA, Heiberg-08, FWHM, FACT, and manual contouring seem to work well regardless of whether the infarct is acute or subacute, or whether it is solid or patchy.

## 4. Discussion

To our knowledge, this study represents the most comprehensive comparisons of all major published in-vivo infarct quantification methods against an ex-vivo validation standard in a porcine model of MI.

Manual delineation agreed well with reference standard and had low inter-observer variability.

The methods EWA, Heiberg-08, FWHM, and FACT all performed on par with manual delineation on IR images compared to T1w reference. On PSIR images EWA and FACT performed on par with manual delineation compared to T1w reference. Both EWA and FACT algorithms have the advantage of working both with IR and PSIR images. Heiberg 08 and FWHM were not designed to work for PSIR images as they implicitly assume that the myocardium is nulled and has a signal intensity that is close to zero and that all intensities are positive.

The FWHM algorithm has previously been reported to be superior to the *n*-SD method [12], [13], [14], which is also supported by the present study. For manual delineation there was one outlier point on IR compared to reference standard. This data point was acquired on the same day of infarction (d0) and had a patchy morphology. It was grossly overestimated by most in-vivo techniques probably due to the acute edema and its morphology. Hence, this data point is an adequate representation of when not only algorithms, but also experienced observers reach their limitations.

Interestingly, none of the quantification methods performed as well as in their original publications. Before addressing this discrepancy, we would like to highlight the challenges and advantages of each algorithm to increase our understanding of potential pitfalls.

### 4.1. LV delineations (all algorithms)

The first step in any infarct size measurement is the delineation of the endo- and epicardial borders of the LV myocardium. It has been shown that the variability in delineation between readers can introduce substantial variability in infarct size [21].

#### 4.1.1. n-SD from remote

**Pros:** The *n*-SD from remote algorithm represents one of the most widely employed techniques to measure the size of MI [3]. **Cons:** Since the number of SD is highly dependent on the noise levels in the image, several multiples of SD have been proposed as thresholds to capture the correct extent of infarction. This makes the *n*-SD from remote method of limited use in multi-center, multi-vendor studies. Based on data from dogs, 2 and 3-SD from remote was recommended [10], [22]. Subsequently 5-SD [23], 6-SD [24] and even 8-SD [13] were reported to be optimal cut-off values. In a meta study on prognostic value on LGE in dilated cardiomyopathy two thirds of the studies (21/34) used visual analysis where the remainder used different n-SD [15]. The literature generally agrees that a 2-SD threshold overestimates the size of the infarction [12], [14], [23], [24]; also supported by our observations in the current study. Furthermore, in a previous study [25] that investigated the n-SD from remote method concluded that “*n-SD from remote method is unreliable for infarct quantification due to high variability which depends on different placement and size of remote ROI, number n-SD, and image signal properties related to the CMR-scanner and sequence used*”. This conclusion is supported by the different multiples of SD that have been proposed in the literature.

#### 4.1.2. FACT

**Pros:** One of the main advantages with the FACT algorithm is that it works with both IR and PSIR images. This study use a re-implementation of the original algorithm developed with close contact with the first author of the original study. The FACT algorithm is now made freely available for research purposes in the software Segment [16]. **Cons:** Inherently the FACT algorithm is an automated version of FWHM and thus shares its weaknesses, including the assumption that selected voxels represents fully infarcted areas which is not always the case (see below).

#### 4.1.3. FWHM

**Pros:** The pros with FWHM is that the method is accurate, validated and relatively insensitive to noise. **Cons:** The FWHM algorithm requires the user to manually define the area of infarction and this maneuver subsequently determines what extent of the infarcted area is included based on its maximum signal intensity. Using maximum intensity value as an anchoring point, the algorithm implicitly assumes that the selected voxel(s) represent a fully infarcted area, which may not be the case in all instances of infarction.

Multiple FWHM implementations exist. The original implementation does not take remote myocardium into account and places the threshold as the half between the highest signal intensity (half maximum) in the infarcted area and the zero level (full width) [8]. This requires that the signal intensity is always positive, which is the case with IR. Negative signal intensity that may occur in PSIR images would lead to an overestimation of infarct size. This conceptual pitfall is most likely an explanation for previously published data [26], where it was reported that FWHM overestimates infarct size in PSIR images compared to IR images. To circumvent this, some groups define the full width not as the maximum value, but as the maximum value minus mean of the remote region. However, this undermines some advantages of the FWHM technique as it requires the definition of a remote ROI. Caution is needed when interpreting data obtained using the FWHM technique, as implementations can vary.

#### 4.1.4. EWA and Heiberg-08

**Pros:** EWA, Heiberg-08 [17], [19], and SI-PIM method [11], [27] operate by weighting each voxel inside the infarct area depending on image intensity. All other methods establishing a signal intensity threshold above which every voxel fully contributes to the measured infarcted volume. These dichotomous approaches do not take partial volume effects inside the infarct into account. The FWHM and FACT algorithm do incorporate partial volume effect, but only as a means of determining the threshold. **Cons:** The cons of Heiberg-08 is that it was not designed to work for PSIR images.

The EWA works by identifying two Gaussian distributions using an expectation maximization algorithm, followed by voxel weighting. In addition to accounting for partial volume effects the advantage of EWA is that no user input is needed. Interestingly, the EWA algorithm is the only thresholding technique employing automated surface coil intensity correction. **Cons**: The cons for the EWA algorithm is that the expectation maximization algorithm assumes that the distribution of normal tissue and scar tissue are both normally distributed, which is an approximation, especially for a perfectly nulled myocardium that more closely follows Rician distribution.

#### 4.1.5. Otsu

**Pros:** The Otsu method has been described to yield excellent correlation when compared to manual infarct delineation [28], [6]. **Cons:** The low performance in this study and in particular for small infarct sizes is likely attributable to the fact that the Otsu method will identify infarcted areas, even when no infarction is present, as the algorithm is designed to identify two populations of signal intensities in any given image.

### 4.2. Imaging with IR vs. PSIR

With IR imaging, correct nulling of the remote myocardium is crucial to generate the desired contrast between the remote and infarcted myocardium. It has previously been estimated that setting the TI only 15 ms before the optimal null TI leads to a change in the contrast-to-noise ratio between remote and infarcted area of 25% [29]. Furthermore, as the optimal TI for nulling represents a moving target as the contrast agent washes out, any threshold to detect infarction may need to change over the course of a multi-slice acquisition. The PSIR technique decreases the importance of selecting a correct TI as it preserves the sign of the magnetization [29], [30].

### 4.3. Imaging at different days post-infarction and patchy vs. solid infarctions

We observed a consistent and significant tendency of all measurement techniques to overestimate acute infarcts (except Otsu). This observation was most pronounced for *n*-SD methods. It has been reported that acute infarctions were overestimated in LGE, possibly due to edema in the salvaged myocardium neighboring the infarction, which results in contrast enhancement [7]. We attribute the observed overestimation, which is particularly pronounced in algorithms that do not compensate for partial volume effects, to the same physiological process.

We speculate that algorithms face similar challenges with partial volume effects and a potential for overestimation for less homogeneous, patchy infarctions that represent interleaved, macroscopically visible mixes between viable and non-viable areas. A tendency toward overestimation, although less pronounced, could also be observed with patchy infarctions (Fig. 5).

### 4.4. Performance compared to original publications

An observation when comparing the data in this work with previously published data on ex-vivo validated infarct algorithms is that the correlation coefficient between in-vivo and ex-vivo infarct measurement is consistently lower than in the respective original work. We attribute this observation to several factors: (I.) This dataset intentionally represents an array of heterogeneous datapoints, including various imaging time-points post MI, a broad spectrum of infarct sizes, different infarct morphologies (solid and patchy), image acquisitions on MRI cameras from two different vendors and from different series of experiments stretching over the timespan of several years. We believe that this heterogeneity better represents the real-world clinical use of infarct measurement algorithms than the conditions reported in the original reports, which can typically be characterized as narrower in some of the above-mentioned dimensions (see Table 1). (II.) An algorithm should ideally be developed as a two-step process where the first step is the design and implementation and a validation step in a pre- defined test set. In the current era of machine learning this is common practice but was less so for hand-crafted algorithms and especially when there is only a very limited number of animals. (III.) The performance of the algorithms is intentionally evaluated with the minimal human interaction to achieve a reading with any given algorithm. Areas that were characterized as infarction by the algorithm, but to the human observer were obvious artifacts were included but may have been excluded manually in the original publications. In addition, there are methodological differences to the original publications such as ischemia times, reperfusion treatment and time of imaging in relation to the ischemic injury.

For clinical or research use, all algorithms should be supervised and, if required, manual corrections should be employed. This study was designed to illustrate the raw baseline performance of each algorithm which represents the most reproducible and least biased measurement. We do not believe that any of the reported observations questions or necessarily invalidates previous reports; however, we believe that this study reveals the real-world challenges that algorithms with minimal human interaction face in a heterogeneous dataset. Furthermore, it underlines the need for critical validation of quantification algorithms and quality control.

## 5. Limitations

This study was conducted in an ischemic porcine model of MI, enabling ex-vivo validation. An animal model of acute infarction in otherwise healthy pigs can only serve as an approximation to the varying and potentially different infarct morphologies that may be encountered in man. We believe that the possibility to perform ex-vivo validation largely outweighs this limitation and is more conclusive than, for example, manual infarct delineations as an in-vivo comparator in a human cohort.

It is known that LGE overestimates infarct size in the acute setting, probably due to acute edema [7]. The present study includes data both in the hyper acute setting (day 0) and data acquired day 3–7, where part of the edema partly has resided. In accordance with previous findings, we found a slight overestimation of infarct size acutely compared to T1w imaging (Fig. 5, top row). Furthermore, a slight overestimation of infarct size by LGE compared to TTC was found in the present study as well (Fig. 2).

This study only investigates quantification of ischemic scar and the results cannot directly be transferred to assessment of non-ischemic scar. Furthermore, mapping data was not acquired, which potentially could add extra information in the patchy infarcts. This study employed high-resolution T1w whole heart scans as an ex-vivo validation standard in favor of the more commonly used TTC staining. This technique has been validated against TTC staining [7], [10] and a subset of the animals in this study (Fig. 2). Undeniably there are methodological differences between T1w imaging and TTC. We believe that the T1w method allows for more accurate calculation of ex-vivo infarct size as it obviates the need for extrapolation between physical slices measuring several millimeters in thickness.

Visible MVO served as an exclusion criterion since only a minority of algorithms can detect and process MVO automatically. In an acute infarction MVO is often present and there need to be mechanisms to incorporate MVO. The algorithms EWA, Heiberg-08, and FACT include automated MVO detection, whereas the other algorithms require manual correction.

## 6. Conclusion

The raw performance of EWA, Heiberg-08, FWHM, and FACT is on par with manual delineation, however, Heiberg-08, and FWHM are not designed for PSIR images. The technique used to measure infarct size should always be disclosed. None of the algorithms perform optimally and a substantial degree of variability remains within each algorithm. Caution should be applied when comparing datasets employing different infarct quantification methods. Manual delineation by experienced readers remains a reliable technique to measure infarct size. Algorithms that perform on par with manual delineation are likely to require little manual interaction and to save time as well as they may serve as a help to inexperienced observers or help align results from multi-center studies.

## Funding

Einar Heiberg is founder of software company Medviso AB, which develops the software Segment that was used in the image analysis.

## Author contributions

**Sascha Kopic:** Writing – review & editing, Writing – original draft, Visualization, Validation, Methodology, Investigation, Formal analysis. **Einar Heiberg:** Writing – review & editing, Visualization, Software, Methodology, Formal analysis. **Henrik Engblom:** Writing – review & editing, Formal analysis. **Marcus Carlsson:** Writing – review & editing. **David Nordlund:** Writing – review & editing, Investigation, Formal analysis. **Robert Jablonowski:** Writing – review & editing, Investigation, Formal analysis. **Mikael Kanski:** Writing – review & editing, Investigation. **Christos Xanthis:** Writing – review & editing, Investigation. **Sebastian Bidhult:** Writing – review & editing, Investigation. **Anthony H. Aletras:** Writing – review & editing, Supervision, Methodology. **Håkan Arheden:** Writing – review & editing, Supervision, Resources, Project administration, Methodology, Funding acquisition, Conceptualization.

## Declaration of competing interests

The authors declare the following financial interests/personal relationships which may be considered as potential competing interests: Hakan Arheden reports financial support was provided by Swedish Research Council. Hakan Arheden reports financial support was provided by Region of Scania, ALF funding. Einar Heiberg reports financial support was provided by Swedish Research Council. Einar Heiberg reports financial support was provided by Region of Scania, ALF funding. Hakan Arheden reports a relationship with Imacor AB, Lund, Sweden that includes equity or stocks. Einar Heiberg and Henrik Engblom reports a relationship with Imacor AB, Lund, Sweden that includes board membership. Einar Heiberg reports a relationship with Medviso AB, Lund, Sweden that includes equity or stocks. All other authors declare that they have no known competing financial interests or personal relationships that could have appeared to influence the work reported in this paper.
